# Wireless Coexistence of Cellular LBT Systems and BLE 5

**DOI:** 10.1109/access.2021.3056909

**Published:** 2021-02-11

**Authors:** SIRAJ MUHAMMAD, MOHAMAD OMAR AL KALAA, HAZEM H. REFAI

**Affiliations:** 1 Center for Devices and Radiological Health, U.S. Food and Drug Administration, Silver Spring, MD 90145, USA; 2 Department of Electrical and Computer Engineering, The University of Oklahoma, Tulsa, OK 74135, USA

**Keywords:** 5G NR-U, BLE 5, coexistence, empirical evaluation, LBT, wireless medical devices

## Abstract

The 2.4 GHz spectrum is home to several Radio Access Technologies (RATs), including ZigBee, Bluetooth Low Energy (BLE), and Wi-Fi. Accordingly, the technologies’ spectrum-sharing qualities have been extensively studied in literature. License-Assisted Access (LAA) Listen-Before-Talk (LBT) has been identified in technical reports as the foundation for the channel access mechanism for 5G New Radio-Unlicensed (NR-U) operating in the 2.4 GHz Industrial, Scientific, and Medical (ISM) band. The introduction of NR-U into this band raises new concerns regarding coexistence of the newcomer with traditional incumbents. This article reports an investigation of BLE 5 and cellular LBT coexisting systems by means of empirical evaluation. The importance of this study stems from that the studied LBT mechanism is indicative of how 5G NR-U would perform in the 2.4 GHz band. Tests were performed in conformity with the American National Standards Institute (ANSI) C63.27 standard for evaluation of wireless coexistence, and results were reported in terms of throughput and interframe delays. In accordance with the standard and under different BLE physical layers (PHYs) and LBT priority classes, three setups were investigated. These pertain to the three tiers of evaluation, which correspond to the criticality of the device under test. Results demonstrated how BLE throughput dropped as the intended-to-unintended signal ratio decreased, and LBT classes exhibited a diminishing effect as the class priority descended. Long Range BLE PHY was found to sustain longer gap times (i.e., delay) than the other two PHYs; however, it showed less susceptibility to interference. Results also demonstrated that low data rate BLE PHYs hindered the LBT throughput performance since they correspond to longer airtime durations.

## I. INTRODUCTION

Equipped with novel advancements in wireless technologies, the Internet of Things (IoT) has ushered in an era of vast connectivity. Numerous devices will be seamlessly connected with each other and exchange various amounts of information to enhance user experience. One pivotal enabler of such a paradigm shift in connectivity, namely Bluetooth Low Energy (BLE), has amassed favorable adoption from many market verticals. According to a Bluetooth SIG market study based on ABI Research forecast, annual Bluetooth-enabled device shipments will exceed 6 billion by 2024 [1]. By virtue of its low power consumption and multiple features, BLE supports various applications in the wearables and smart infrastructure (home, buildings, cities, etc.) industries—from smartwatches and fitness trackers to health sensors and medical innovations. Accordingly, the Bluetooth SIG has been regularly updating BLE specifications. For example, the fifth version [2] introduced new features to the physical layer (PHY), such as high data rate and coding schemes to permit long-range communication links. Further enhancements were announced in revisions 5.1 and 5.2 (e.g., direction finding and audio streaming, respectively [3], [4]).

Nonetheless, mobile broadband networks have been challenged with accommodating a colossal amount of data in the near future. According to an Ericsson report, by 2022 smartphones are forecast to generate more than 60 exabytes of data per month [5]. As a result, unlicensed spectrum serves as an opportunity for mobile network operators to accommodate the increasing demand. In light of this, the 3rd Generation Partnership Project (3GPP) has facilitated the operation of fourth-generation standard (4G) Long-Term Evolution (LTE) in the 5 GHz unlicensed Industrial, Scientific, and Medical (ISM) band by means of License-Assisted Access (LAA) technology. This proposition has received much attention from the industry and academic institutions, primarily because it implies coexistence with incumbent technologies, especially widely used Wi-Fi devices [6]. LTE-LAA was established using a channel random-access scheme known as Listen-Before-Talk (LBT), which addressed compliance with spectrum etiquette set forth by regulators (e.g., European Telecommunication Standard Institute (ETSI) [7]), as well as fairness with Wi-Fi [6], [8], [9].

Recently, early-stage development of 5G cellular communication has been formulated to consider unlicensed access (i.e., 5G New Radio-Unlicensed (5G NR-U)). 3GPP’s TR 38.889 [10] technical report identifies LBT used in LTE-LAA as a baseline for use in the 5 GHz unlicensed band, as well as the design start point for the newly regulated 6 GHz band [11]. Notably, TR 38.889 includes the 2.4 GHz band within the scope of NR-U operations, unlike previous considerations for LTE-LAA [12]. However, the 2.4 GHz ISM spectrum is already crowded with multiple incumbent technologies (e.g., Bluetooth, ZigBee, and IEEE 802.11b/g/n/ax). In addition, IEEE has formed a study group to discuss a potential amendment—IEEE 802.11be Extremely High Throughput (EHT)—that will build on 802.11ax and target all sub-7 GHz unlicensed spectrum (i.e., 2.4, 5, and 6 GHz bands) [13], [14].

This work is motivated by the need to understand the potential impact arising from the introduction of novel cellular LBT systems in the 2.4 GHz ISM band and its wireless coexistence with longtime incumbent, BLE. The LBT-based LTE-LAA and Wi-Fi wireless coexistence in the 5 GHz band has been studied extensively in literature; observations could be extended for insight on corresponding operations in the 2.4 GHz spectrum. However, the channel access protocol of BLE 5 significantly differs from that of Wi-Fi’s, rendering these observations inconclusive and motivating separate and extensive evaluation of BLE 5 and LBT coexistence. Hence, the study of BLE 5 with cellular LBT is necessary to characterize degradations of the BLE key performance indicators which could lead to adverse consequences, especially those related to wireless medical equipment. If medical devices incorporate RF wireless technology, the FDA recommends addressing the risks associated with using such devices in proximity to other wireless in-band sources through coexistence testing in their premarket submission [15]. The work highlighted in this article contributes to the understanding of wireless coexistence of BLE medical devices and general equipment operating with LBT cellular systems in the same environment.

### A. CONTRIBUTION

To bridge the literature gap on LBT and BLE coexistence in the 2.4 GHz ISM band, this article presents an empirical evaluation of wireless coexistence among BLE 5 systems and systems employing a cellular LBT channel access scheme in the 2.4 GHz band. This study offers an indication of how 5G NR-U would behave when deployed in environments where BLE devices are used (e.g., hospitals, homes, clinics). The interplay of different LBT channel access priorities and BLE physical layers was assessed, and the mutual impact is reported in terms of normalized throughput and interframe timings as a measure of delay. By doing so, the study characterized and explored the boundaries of operation for BLE 5 when coexisting with LBT-based networks in the 2.4 GHz ISM band as the underlying wireless connectivity for wearable medical devices. The American National Standards Institute (ANSI) C63.27 [16] radiated anechoic chamber test method for evaluating wireless coexistence was adopted in the experimental setup. The experimental findings can be used to augment the standardized ANSI C63.27 coexistence testing and inform the design, development, and deployment of co-located LBT-based and/or BLE 5-based wireless medical equipment. To the best of the authors knowledge, this is the first work to report on these aspects.

The balance of this article is organized as follows. Section II surveys the literature on the coexistence of Bluetooth, Wi-Fi, and LTE-LAA. Section III expounds the LBT mechanism and highlights some of the new features of BLE 5. Section IV details the setup employed for this experimental study. Results are presented in Section V, and a discussion follows in Section VI. Section VII concludes this article.

## II. RELATED WORK

Bluetooth and Wi-Fi have been longtime incumbents of the 2.4 GHz ISM band. Consequently, many works have addressed coexistence issues between the two. Howitt *et al.* [17] presented an empirical analysis of coexistence relative to the IEEE 802.11b network and an early version of Bluetooth. The authors’ investigation evaluated the interference power at which a retransmission is required. In [18], researchers evaluated the impact of Bluetooth 2.1 on the accuracy of Wi-Fi positioning algorithms. Adaptive Frequency Hopping (AFH) was found to decrease the adverse effect on positioning performance. Analytical PHY analysis of BLE, 802.15.4 (ZigBee), and 802.11b (Wi-Fi) was presented in [19]. Expressions for packet error rates were derived as a function of distance provided by path-loss models and symbol error rates. PHY models of affected technologies were used to calculate the symbol error rate as a function of signal to interference ratio facilitated by path-loss models. However, analytical expressions did not capture the behavior of medium access control (MAC) layer mechanisms, such as Carrier Sense Multiple Access (CSMA) and AFH. Instead, an experimental study was conducted to assess their behaviors. Performance of intra-vehicular BLE-based and ZigBee-based wireless sensor networks was investigated in [20] in the presence of classical Bluetooth, as well as Wi-Fi interference. Results indicated Wi-Fi degrades both sensor network performances, although BLE-based networks demonstrated better resilience and robustness than ZigBee-based networks. Classical Bluetooth was evaluated empirically in [21] for music streaming and hands-free calling under interference from three 802.11n networks employing non-overlapping channels (i.e., 1, 6, and 11). The study demonstrated the criticality of classical Bluetooth channels 71 through 78 to sustain connectivity in a Wi-Fi-crowded environment. Results also indicated that a hands-free calling profile is more susceptible to interference than music streaming due to lack of retransmissions in the former. An extension to this work in the automotive domain when considering the mobility effect was reported in [22]. Bronzi, *et al.* [23] investigated BLE with one, two, and three Wi-Fi access points occupying the 2.4 GHz band, as well as in a vehicular communication setting. In [24], Ancans, *et al.* assessed the throughput of a BLE 5 device under test (DUT) in the presence of a single-channel Wi-Fi network with up to four BLE interferers in the environment. Several parameters of the DUT were investigated (e.g., connection interval, PHY layer, and packet size). Results suggested that a 1M BLE PHY layer is more robust to interference than the newly introduced 2M PHY in BLE 5, despite the fact that 2M PHY offers higher application throughput. Authors also reported the effect of multiple BLE links on a single BLE DUT as a function of its connection interval. Results revealed that application throughput deteriorates as the number of BLE devices sharing the spectrum increases, and the DUT becomes more susceptible to interference with longer connection interval, a behavior also observed in this study under LBT interference and discussed in Section V-A. In [25], a performance comparison of the three PHY modes of BLE 5 was presented; trade-offs, with respect to energy consumption, link reliability, and throughput were evaluated. Robustness to Wi-Fi interference was considered with only a single channel running 802.11b protocol. Another empirical study reported in [26] compared BLE 5 operating in Coded PHY (i.e., Long Range) with its precursor (i.e., BLE 4) in an indoor and outdoor setting in terms of communication range and throughput.

With LBT serving as the channel access scheme of LTE-LAA and operating in the 5 GHz ISM band, the technology was investigated due to concerns of coexistence with incumbent radio access technologies (RATs), like Wi-Fi. In [27], LAA LBT and Wi-Fi Enhanced Distributed Channel Access (EDCA) were modeled as a Markov chain, and an approximate closed form for the probability of successful transmission was derived. Coexistence as a function of the number of LAA/Wi-Fi nodes was assessed by means of achieved throughput, average contention delay, probability of successful transmission, and collision. Another analytical model employing energy detection threshold and its effect on throughput performance was reported in [28]. An empirical study on co-channel coexistence between Wi-Fi and LTE-LAA was published in [29]. Channel occupancy of LTE-LAA was measured for a varying combination of Modulation and Coding Schemes (MCS) and achieved throughput for both networks was tracked during coexistence. Fairness between the two systems was studied in [8] according to 3GPP’s definition and other notions of fairness, such as proportional fairness. With LBT being the de facto channel access scheme for 5G NR-U, homogeneous coexistence for such networks without interference from other RATs was investigated in [30] for various channel access priorities. Similar work was reported in [31] for a Wi-Fi/LTE-LAA scenario where two types of services were simulated, namely real-time (video streaming) and non-real-time (FTP), in a dense indoor environment.

## III. TECHNOLOGY OVERVIEW

### A. LISTEN-BEFORE-TALK (LBT)

3GPP had two versions of LBT proposed in their technical reports and specifications. The first was introduced in TR 36.889 [12] in 2015 and was not compliant with regulations set forth later by ETSI in 2017. In EN 301 893 [7], ETSI formally detailed the LBT mechanism that was later adopted by 3GPP in TS 36.213 [32] in 2017 for making LTE-LAA amenable for deployment in the unlicensed spectrum. Despite this, the majority of research disseminated on this topic relied on the old, non-standardized version of LBT.

**FIGURE 1.**
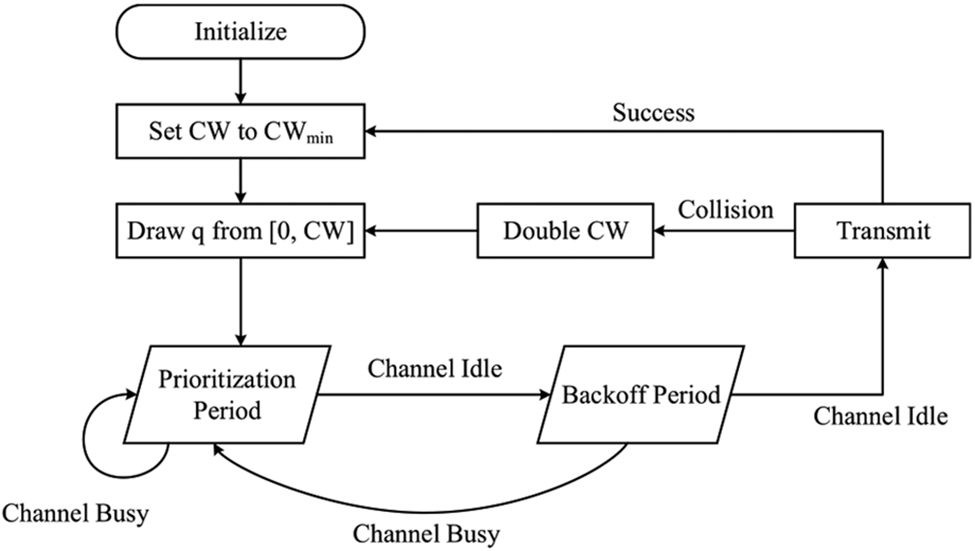
A high-level flowchart demonstrates the LBT procedure for Frame Based Equipment, as stipulated in ETSI standard.

The mechanism is purposed to detect various in-band RATs transmissions and refrain from interfering with them while the detected power is above a predefined threshold. Additionally, the ETSI standard defines four sets of channel access parameters assigned to data packets that determine the contention behavior on the channel and the duration for which they are allowed to endure. Accordingly, high priority packets are more likely to gain access but must have a shorter duration. Table 1 lists the parameters of these classes, with 4 being the highest priority class and 1 the lowest. Channel Occupancy Time (COT) is the maximum time not to be exceeded by nodes when utilizing the channel. The value of P0 and contention window sizes are given in terms of the number of observation slots. Note that the standard allows Class 1 and 2 to increase their COT to 8 ms, given that pauses of at least 100 *μ*s are inserted during transmission. The LBT procedure starts with a waiting period equal to 16 *μ*s, referred to as Short Inter-Frame Spacing (SIFS), followed by the prioritization period (P0 in Table 1), the value of which is determined by the packet class. P0 is a Clear Channel Assessment (CCA) period used to determine channel state (e.g., idle or busy) and differentiates between frame types; low priority frames wait for longer P0 periods. When both SIFS and P0 expire without detecting channel activities registered above the Energy Detection (ED) threshold, the equipment may start the contention process (i.e., each observation slot in SIFS and P0 must pass a CCA). Subsequently, the backoff mechanism starts by initializing the channel access parameters, which are also determined by the priority class of traffic. This comprises setting the contention window *CW* to its minimum value *CW*_*min*_ and drawing a random number *q* between 0 and *CW* – 1. The value of *q* is the number of timeslots the equipment needs to implement CCA. During a single observation slot, the channel is considered occupied if transmissions were detected with a level above the ED threshold, in which case, the LBT procedure starts anew with the SIFS period. Otherwise, the value of *q* is decremented by exactly one. If *q* reaches 0, the device gains access to the channel and may transmit. Afterwards, if a transmission fails, the device may attempt a retransmission subsequent to adjusting its contention window size. *CW* is set to 2^*i*^*CW*, where *i* is the backoff stage (i.e., the contention window is doubled until it reaches the frame’s maximum value *CW*_max_. Fig. 1 illustrates this procedure in a flowchart (See Annex F in [7] for an expanded chart).

Although the design of LBT is similar to EDCA’s which makes LTE-LAA and NR-U on a par with Wi-Fi in the unlicensed spectrum, parameters of the two mechanisms are different, which may give LBT-enabled equipment an edge over Wi-Fi. For instance, LBT frame priority classes support smaller P0 and *CW_max_* values than EDCA’s for some classes and, consequently, LBT-enabled devices are expected to capture the channel faster than Wi-Fi. In addition, the COT values are larger for class 1 and 2 in LBT (6 ms and can extend to 8 ms with 100 *μ*s pauses), whereas COT values for EDCA classes range between 2.528 ms and 6.016 ms [33]. Moreover, EDCA mandates that data packets are assigned priorities relevant to the type of payload being sent (Background, Best Effort, Video, and Voice), while LBT does not impose a similar requirement on transmitting devices and priorities are assigned regardless of the payload type.

### B. BLUETOOTH LOW ENERGY (BLE) 5

BLE 5 has ushered in major improvements—some of the most relevant are highlighted below.

#### 1) PHY MODES

**FIGURE 2.**
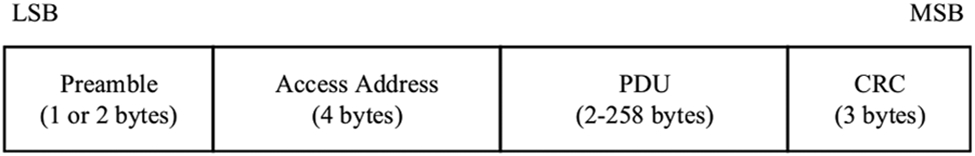
Link layer packet format for BLE uncoded 1M and 2M PHYs.

Prior to version 5, BLE utilized a single PHY with a symbol rate of 1 mega symbol per second (Msym/s). This remains the default setting in the new specifications and serves as a mandatory option all BLE 5 devices are required to support. However, new applications are emerging with requirements for higher data rates and low-power wireless communications (e.g., firmware upgrades delivering new functionalities and security improvements or uploading accrued sensor data to a companion device, such as a smartphone or a PC). A similar trend has been taking place in the health care industry, such as remote multi-lead electrocardiogram (ECG) devices [34], [35]. Hence, the Bluetooth SIG has introduced a 2 Msym/s PHY (referred to as 2M PHY), promising twice the data rate as the original 1M PHY. The link layer packet format for both modes is the same, as depicted in Fig. 2. Depending on the utilized PHY, the preamble can be 1 byte (1M PHY) or 2 bytes (2M PHY) long to maintain an 8 *μ*s duration, followed by 4 bytes for the access address, 2 to 258 bytes of payload in the Packet Data Unit (PDU) field, and 3 bytes for CRC checksum and error detection. Both PHY modes utilize Gaussian Frequency Shift Keying (GFSK) modulation; however, since higher symbol rate might produce inter-symbol interference (ISI), 370 kHz frequency deviation is used in 2M while 185 kHz continues to be used in 1M PHY. No coding scheme is employed and, therefore, error correction is not possible with these two physical layers. Since 2M PHY offers double the speed of the original 1M PHY, airtime used for transmitting a given amount of data is reduced, which in turn improves power consumption and spectral efficiency.

**FIGURE 3.**
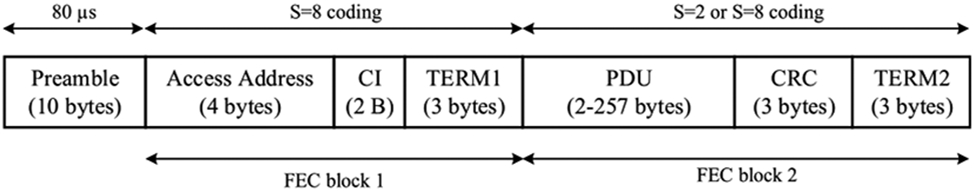
Link layer packet format for BLE Coded PHY (LR).

When long-range communication links are advantageous (or reliability and robustness against interference is desirable), a third physical layer introduced by the Bluetooth SIG in version 5 of their core specification plays a convenient role. Long Range (LR)—technically known as Coded PHY—extends the feasible communication range of BLE beyond the typical 50-meter marker to achieve more than 1 km in distance, as reported by Nordic Semiconductor [36]. LR PHY makes use of Forward Error Correction (FEC) with symbol coding of 2 (S2) or 8 (S8) symbols per bit. Since this mode uses a physical rate of 1 Msym/s, resulting data rates are reduced to 500 kbps and 125 kbps for S2 and S8, respectively. A different link layer packet format is employed in LR PHY, as illustrated in Fig. 3. Each packet comprises a preamble, FEC block 1, and FEC block 2. The preamble is 10 bytes long and not coded to allow cross-PHY detection. FEC block 1 consists of a 4-byte access address, coding indicator (CI) which denotes the coding scheme used in the following FEC block 2 (i.e., S2 or S8), and 3-byte termination field (TERM1). FEC block 1 is always coded with 8 symbols per bit regardless of the coding configuration of the packet (CI field). FEC block 2 contains the PDU, which is 2-257 bytes, 3-byte CRC, and a second 3-byte termination field (TERM2). The second FEC block is coded with either S2 or S8 scheme. It is worth noting that although Coded PHY exhibits higher reliability than 2M and 1M PHYs by virtue of FEC, it also incurs lower throughput and higher power consumption due to larger packet sizes that lead to longer radio-on times. Table 2 summarizes the three physical layers.

#### 2) CHANNEL SELECTION ALGORITHM #2

**FIGURE 4.**
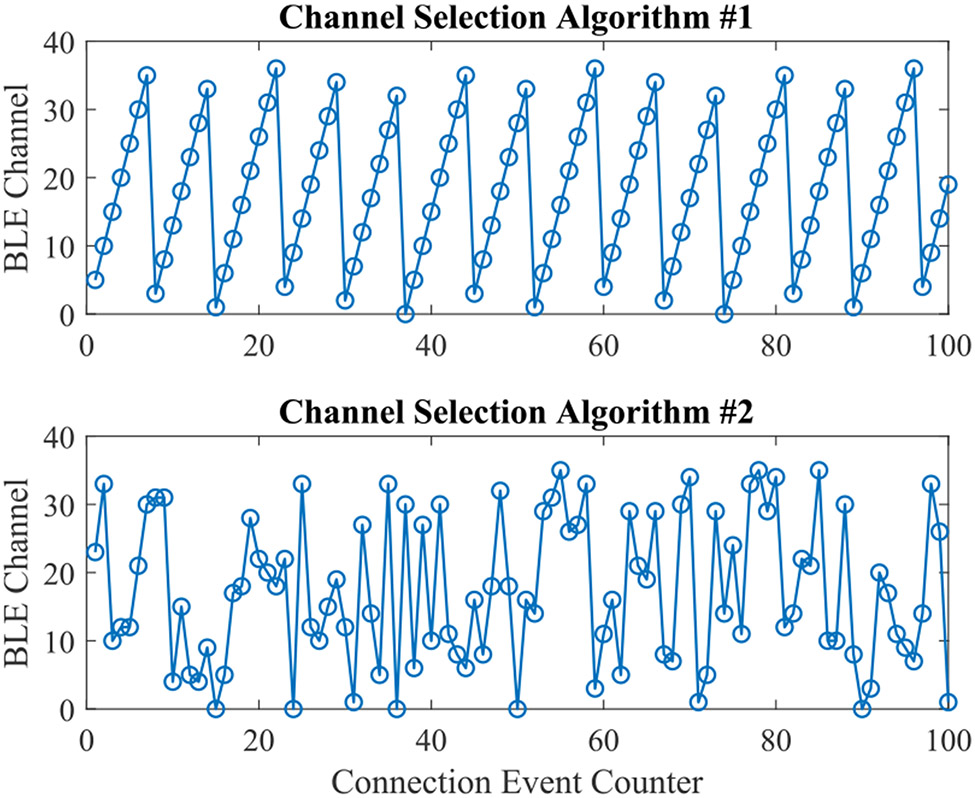
Channel hopping pattern of BLE channel selection algorithms #1 (top) and #2 (bottom) over 100 connection events.

**FIGURE 5.**
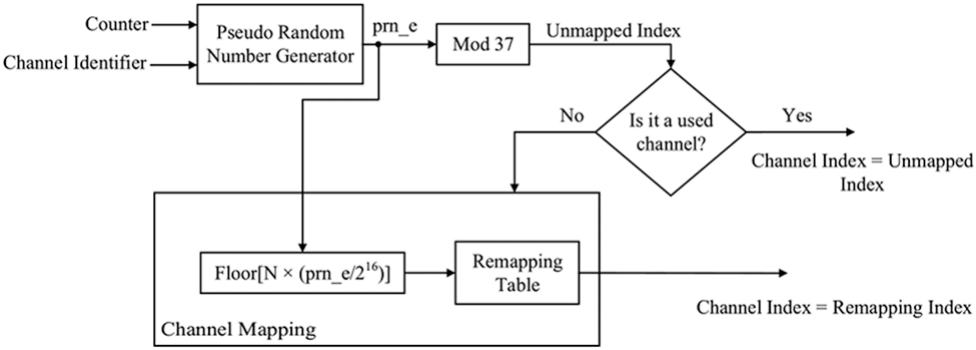
Block diagram of channel selection algorithm #2 introduced in BLE version 5.

Bluetooth technology employs Adaptive Frequency Hopping (AFH) spread spectrum to maneuver in-band interference. The link layer classifies the RF channels into used channels and unused channels, creating a channel map that is applied during data transmission [2]. Before BLE 4, the channel sequence generation process utilized an algorithm that produced incremental, easy-to-track hopping patterns that were suboptimal at avoiding interference given that hopping was not random, and packets of the same connection event would use the same channel [37]. The new channel selection algorithm (i.e., CSA #2) is a more complex method and generates harder-to-track pseudo random sequences. In Fig. 4, we compare the hopping pattern of the two CSAs across 100 connection events simulated in MATLAB using the Communication Toolbox™ Library [38]. CSA #2 employs a Pseudo Random Number Generator (PRNG) engine requiring two 16-bit inputs, a channel identifier, and a counter that increments with each connection event. The connection identifier is fixed for any given connection and is calculated by a bitwise XOR operation of the upper two bytes with the lower two bytes of the access address. PRNG output serves as the channel index for the next connection event. Since the link layer might classify some channels as unused, the generated channel index is remapped if it falls within the excluded channel list. Fig. 5 depicts a high-level block diagram of the procedure. Readers interested in more details are referred to Section 4.5.8, Vol. 6, Part B in [2].

## IV. EXPERIMENTAL SETUP

The test setup utilized in this work was devised according to ANSI C63.27 standard [16] in which three-tier evaluations are specified to address different levels of criticality of the device under test. Consequence of failure in the functional wireless performance (FWP) with regard to possible lack of coexistence determine the evaluation tier. In the example of wireless medical devices, the risk assessment and mapping to ANSI C63.27 evaluation tiers can be done using the Association for the Advancement of Medical Instrumentation (AAMI) Technical Information Report (TIR) 69 for risk management of radio-frequency wireless coexistence for medical devices and systems [39].

**FIGURE 6.**
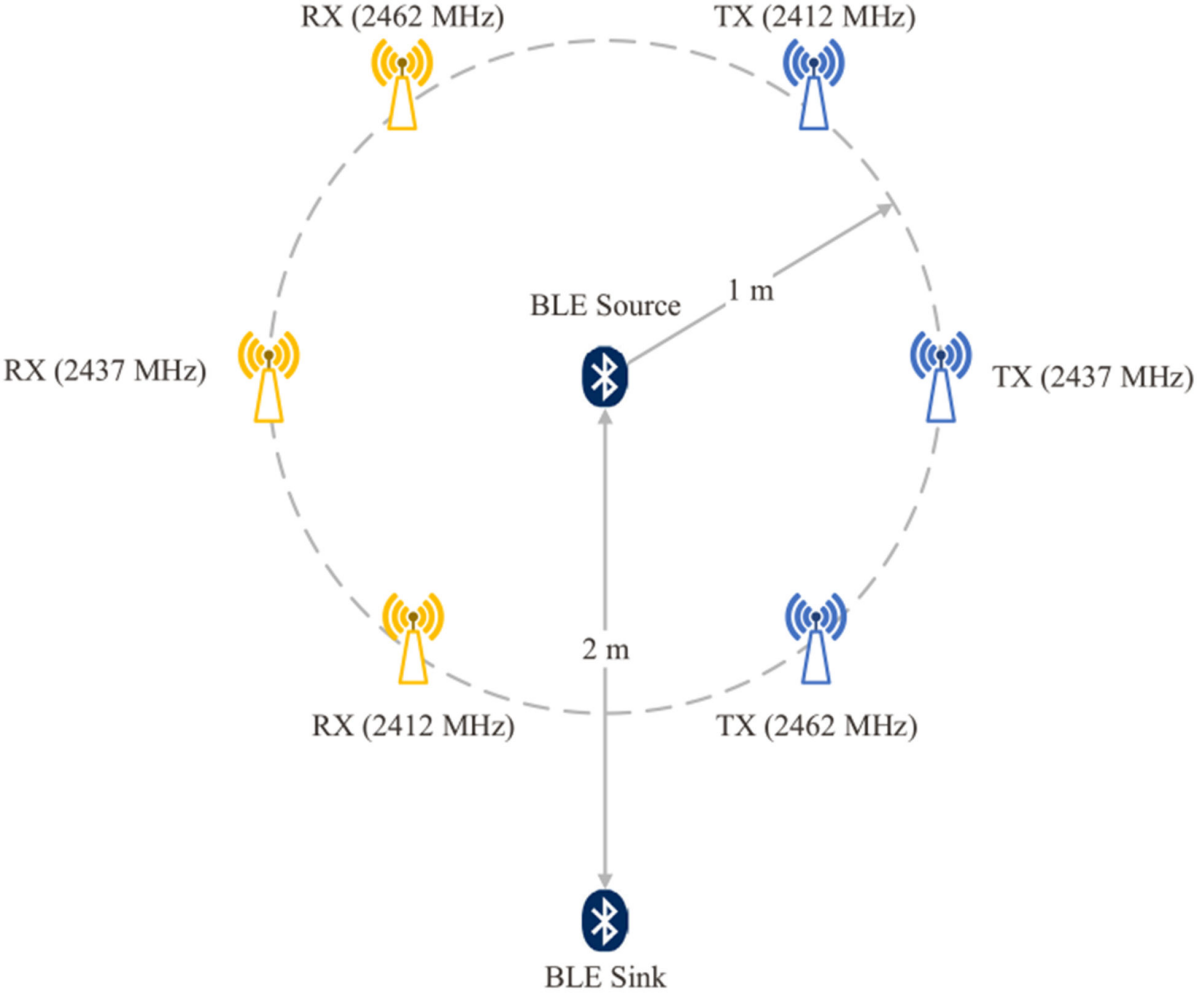
The experimental setup of the coexistence test illustrating the arrangement of BLE nodes and the three LBT pairs with center frequencies 2412 MHz, 2437 MHz, and 2462 Mhz.

In the considered test setup, LBT network is treated as the unintended source of interference, while BLE network acts as the DUT, in other words the intended signal. The C63.27 standard provides band-specific test guidance for common RATs along with recommendations on the choice of unintended signals for testing. Described guidance for BLE was followed, with the exception that the unintended IEEE 802.11n signals were replaced with signals representative of cellular LBT in all evaluation tiers. Tier 1 is the most extensive level of evaluation, where a rigorous set of unintended signals challenge the FWP of the DUT. Accordingly, three 20 MHz non-overlapping LBT channels were imposed on the DUT. Tier 2 is concerned with coexistence evaluation with lower level of rigor than Tier 1. In our setup, the unintended signals occupied 20 MHz channels that correspond to Wi-Fi channel 1 (2412 MHz) and 11 (2462 MHz). DUT was exposed to the minimum number of unintended signals in Tier 3, and, therefore, one LBT channel was centered at 2437 MHz (i.e., corresponding to Wi-Fi channel 6). LBT nodes were placed in a circular arrangement around the BLE source node with radius of 1 m to ensure equal power level at the DUT from all three channels of interference, per ANSI C63.27 Annex A recommendations. Other test layouts can be used depending on the DUT’s functionality and the intended environment (e.g., line of sight, non-line of sight). BLE sink node was placed 2 m from the source outside the circle, as depicted in Fig. 6. According to BLE specifications, each data packet must be acknowledged by the receiving device by sending an empty packet. Furthermore, we note that BLE does not perform a clear channel assessment (CCA) like it is typical in Wi-Fi and LBT systems. Accordingly, for our specific scenario where both the source and the sink were configured with the same TX power, swapping the roles of BLE devices is expected to have a similar outcome since the effect of a dropped acknowledgment packet is similar to that of a dropped data packet, as discussed later in Section V. Tests were conducted in a semi-anechoic chamber to eliminate uncontrolled interference, and an NI PXIe-1071 [40] spectrum analyzer was used to measure power levels.

Three NI USRP 2943R [41] devices implementing the LBT mechanism using LabView were deployed as the LBT networks. The setup utilized NI LAA implementation which was based on their LTE Application Framework [42] and extended to support LBT channel access scheme, details can be found in [43]. LBT and PHY were synthesized on the FPGA to account for critical timing requirements. Modifications to the LAA LBT code provided in [43] were made to resemble the ETSI-compliant LBT detailed in Section III-A and incorporate SIFS and P0 durations pertaining to the four-class priorities. Parameters such as backoff window size and transmission opportunity (TXOP) duration (or channel occupancy time [COT]) were made accessible in the user interface to change channel access priority in run-time, as per Table 1. Exponential backoff was not supported by the used devices. Accordingly, only maximum backoff window size was considered. [29] demonstrated that MCS has a negligible effect on channel utilization and that the highest MCS introduces the greatest impact on both coexisting systems. Consequently, to account for worst-case scenarios, the highest MCS was selected in all priority classes. Each node was set in an RF loop-back configuration. An internal loop-back allows the calculation of cyclic redundancy check (CRC) of received data on the physical downlink shared channel (PDSCH) over the air. Achieved throughput was monitored and recorded for each test.

A Nordic Semiconductor nRF52480 dongle kit was used as the BLE network [44]. The throughput example provided in the Software Development Kit was modified to support all three BLE PHYs (i.e., legacy 1M, high data rate 2M, and coded Long Range [LR]) and different levels of transmission powers. The BLE source sent a configurable amount of random data (e.g., 1 MB) to the sink node and reported achieved throughput at the end of the test. During transmission, a BLE sniffer placed next to the sink collected and reported performance indicators (e.g., packet error rate and retransmission rate, as well as histogram of the utilized channels). Configuration parameters are listed in Table 3.

The use of 2.4 GHz ISM band for future LBT-based 5G NR-U networks might be targeted towards low traffic profiles, compared to the more accommodating Unlicensed National Information Infrastructure (UNII) bands at 5 GHz and the newly regulated 6 GHz unlicensed spectrum. Nevertheless, from an exploratory perspective, ANSI C63.27 recommends investigating the coexistence parameters (i.e., frequency, range, and time) to identify the characteristics of the DUT’s failure modes. Hence, the unintended LBT nodes were operated in full buffer mode as a worst-case scenario that attempts to generate the highest channel utilization. To increase the chances of exposing weaknesses and further discover failure characteristics of coexisting RATs, a wider set of testing scenarios were examined by considering various intended-to-unintended (I/U) signal ratios [16]. Using the spectrum analyzer, the signal levels of the companion BLE device and the interferer LBT device were measured at the DUT (i.e. the BLE source at the center of the circle). While the DUT is off, the unintended signal was measured with a max hold detector over 2 MHz channel bandwidth, as observed by the BLE device. Subsequently, the intended signal was measured by reversing the BLE roles and transmitting from sink to source. When the unintended signal was measured at −48 dBm and the BLE transmission power changed between 8 dBm and −12 dBm (for the source and the sink), a range of I/U ratios between 1 dB and −19 dB was noted.

For each evaluation tier, LBT nodes were configured to transmit packets pertaining to one of the four access priority classes. For each class, the BLE network was tested in one of the PHY modes—2M, 1M, or LR. Transmission power of the BLE network was varied between 8 dBm and −12 dBm with 4 dB step. Each test was repeated five times to ensure repeatability. Thus, a total of 1080 test vectors were collected.

## V. EMPIRICAL RESULTS

In this section results of the three tiers of evaluation are presented, and a discussion follows in the subsequent section. It should be noted that the results indicate the expected coexistence behavior of cellular LBT and BLE 5. However, unique device implementations across the open system interconnection (OSI) layers warrants individual evaluation when needed as detailed in ANSI C63.27.

### A. IMPACT OF LBT ON BLE 5 PERFORMANCE

**FIGURE 7.**
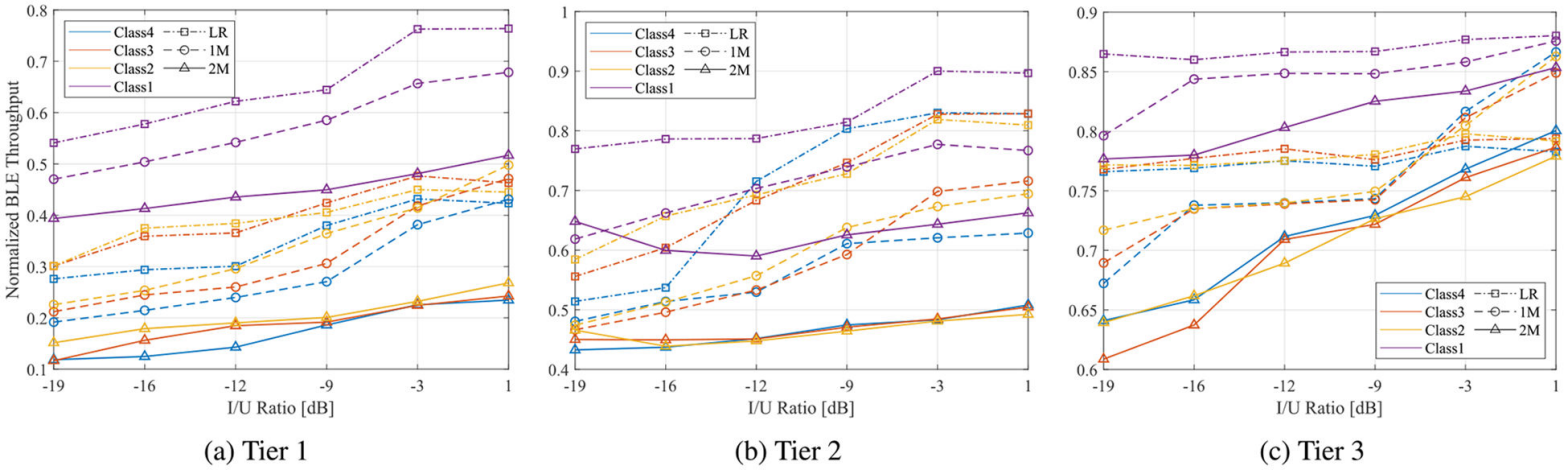
Normalized BLE throughput under LBT interferers of class 1, 2, 3, and 4.

Tier 1—with three LBT interferers centered on frequencies 2412 MHz, 2437 MHz, and 2462 MHz—poses the biggest challenge to the BLE 5 network since LBT unintended signals utilize 60 MHz of the 2.4 GHz band. All three channels are used to transmit packets of the same priority class (e.g., class 1, 2, 3, or 4). As the priority increases, the contention window size and channel occupancy time decrease. High priority nodes capture the channel faster than a lower priority, although they utilize the channel for smaller durations. The effect of these signals on the normalized throughput of the 2M, 1M, and LR BLE PHYs acting as a function of the I/U ratio is shown in Fig. 7a. Although all BLE PHYs experience reduction in their throughput to less than 50%, 2M sustains the highest impact under all priority classes of interferer. Though measurements indicate that LBT classes 4, 3, and 2 tend to have a decreasing effect on the achieved BLE throughput, their lines are clustered and their impact is relatively small under the same BLE PHY—except for class 1, which has the least impact on all physical layers. The figure accentuates the resilience of low data rate PHYs to interference noted in Section III-B which comes, however, at the expense of throughput; the maximum achieved by LR and 1M PHYs in baseline without interference is 26 Kbps and 226 kbps, respectively, compared to 340 kbps for 2M PHY. Curves in Fig. 7 are normalized with respect to these maximum values.

**FIGURE 8.**
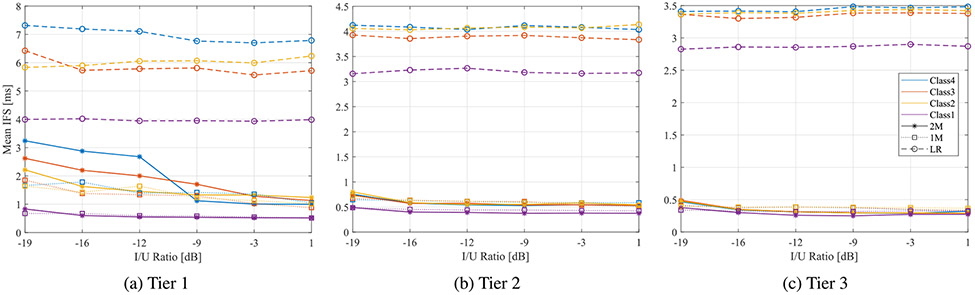
Mean IFS durations of BLE PHYs in three tiers as a function of the I/U ratio.

Since various applications depend on different key performance indicators, other metrics were evaluated. Interframe spacing (IFS) durations between successful packet transmissions were analyzed using sniffer capture files as a measure of latency. Fig. 8a compares calculated IFS for BLE 2M and LR physical layers under the four LBT priority classes and as a function of the I/U ratio. Interestingly, the figure suggests that the average gap time between successfully received packets was higher for LR PHY than 2M PHY. Additionally, the figure demonstrates that IFS for BLE 2M PHY increased as the I/U ratio decreased, whereas LR PHY does not exhibit a similar relationship, indicating that IFS is less sensitive to interference for that physical layer. BLE 1M PHY showed a similar trend to 2M PHY, with IFS values between 1 and 2 ms.

BLE throughput performance in tiers 2 and 3 was similar to tier 1; normalized throughput dropped as the I/U ratio decreased, and differences among LBT priority classes within the same BLE PHY were negligible. However, Fig. 7b and 7c illustrate that 1M PHY performed better than LR in tier 3 when I/U was above −3 dB. Furthermore, LR achieved throughput appeared to flatten as a function of I/U in tier 3, indicating less susceptibility to interference in relaxed conditions. Tiers 2 and 3 in Fig. 8b and 8c demonstrate similar behavior to tier 1 in terms of IFS durations with lower impact.

**FIGURE 9.**
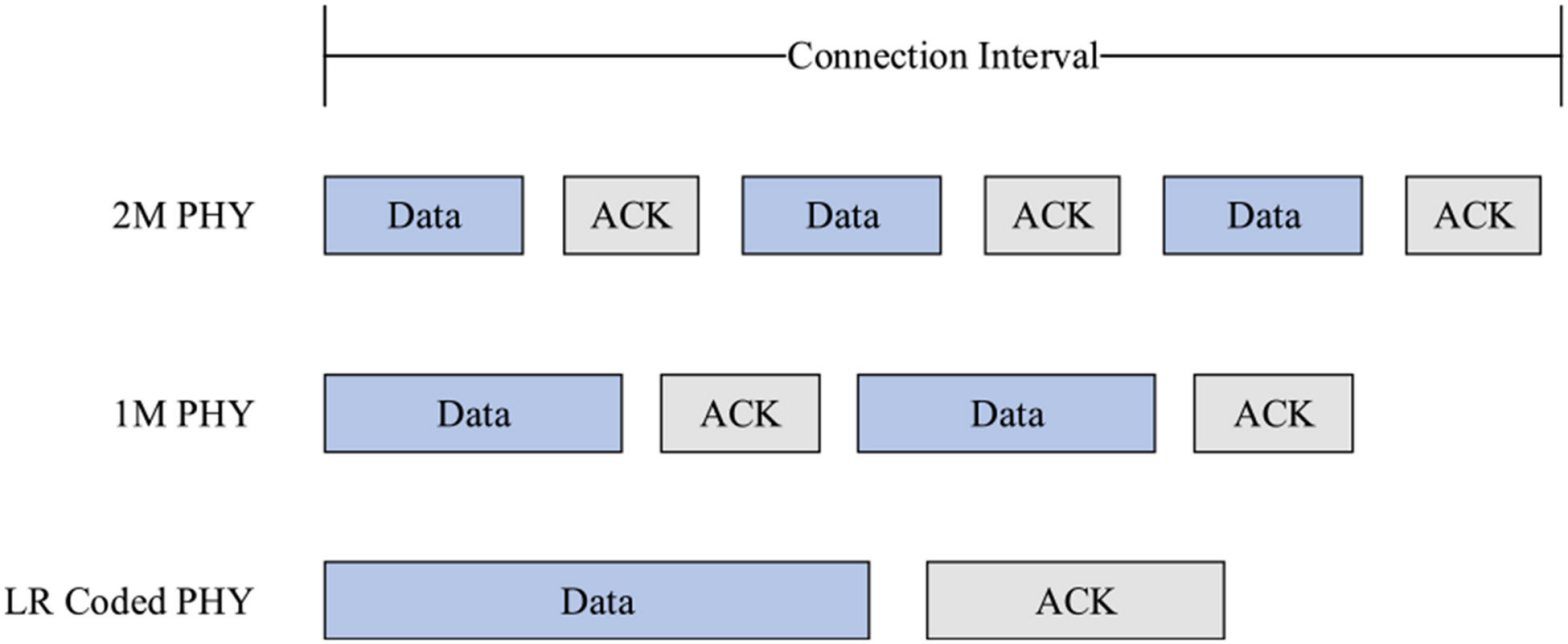
An example of the number of packets that can be sent during one connection event for 2M, 1M, and LR PHYs.

This observation of elevated implications on IFS in LR PHY and its resistance to I/U ratio can be explained in the context of connection interval and packet duration. Connection interval is defined as the time between two BLE connection events that involves data transfer between two BLE devices. The number of packets that can be sent during one connection event depends on the physical layer agreed upon at the beginning of a connection; therefore, the time spent to transmit a given amount of data using LR PHY is longer than 1M and 2M PHYs. Consequently, the number of packets sent in one connection event is less for LR compared to the other two PHYs, as illustrated in Fig. 9.

If a packet (data or ACK) is not received or dropped (i.e., a situation may occur due to noisy channel or interference from other incumbents’ transmissions), the delay between two successive received packets increases which also reduces the achieved throughput. Notably, when interference causes the first frame of the connection event to be dropped, transmitter must wait for the next connection event to send its packets. The repercussions of such situations are higher on LR PHY than 2M and 1M since the number of packets that can be sent are fewer within the same connection interval. On the other hand, LR PHY implements Forward Error Correction (FEC), which allows it to recover some erroneous bits on the receiver side without the need to retransmit the packet. Hence, the mean IFS is less susceptible to I/U ratio and more so to time activity of the interfering system; this is manifested by the effect of the access priority class of LBT, as shown in Fig. 8. One can also note that class 2 has higher impact than class 3 across the three tiers. This is attributed to the fact that class 2 exhibits longer channel occupancy time (6 ms) compared to 4 ms for class 3. Class 4 effect is comparatively less than the other three classes due to its exponentially large maximum contention window size.

Findings discussed in this subsection agree with similar studies found in literature and go beyond what was presented by addressing all physical layers of BLE under single- and multi-channel interference. Ancans *et al.* [24] reported that BLE throughput using 2M and 1M PHYs was reduced by approximately 30% when subjected to a single-channel interference from an unspecified variant of Wi-Fi protocol, configured on channel 1 (2412 MHz). Though the authors did not report the power configuration of BLE and Wi-Fi, it appears from their figures (6 and 7 in [24]) that I/U was around −8 dB. Likewise, results of Tier 3 scenario suggest that 2M and 1M PHYs sustain comparable reduction under class 4, 3, and 2 LBT interferers. Spörk *et al.* [25] also reported similar work with a single-node 802.11b interference centered on channel 6 and configured to transmit a 1500-byte long packet every 10 millisecond. BLE connection was configured to only use BLE data channels (12 to 19) overlapping with Wi-Fi’s channel 6. Results showed confirm that LR PHY provides better reliability under interference compared to 2M and 1M PHYs.

### B. IMPACT OF BLE 5 ON LBT PERFORMANCE

**FIGURE 10.**
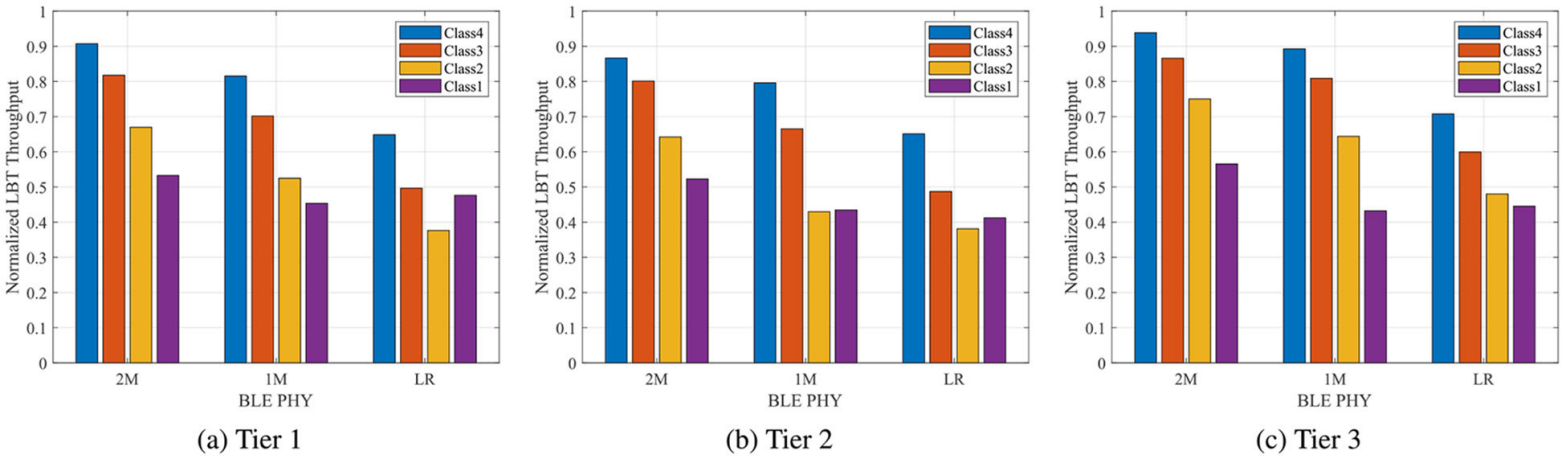
Normalized LBT throughput in the evaluation of three tiers.

**FIGURE 11.**
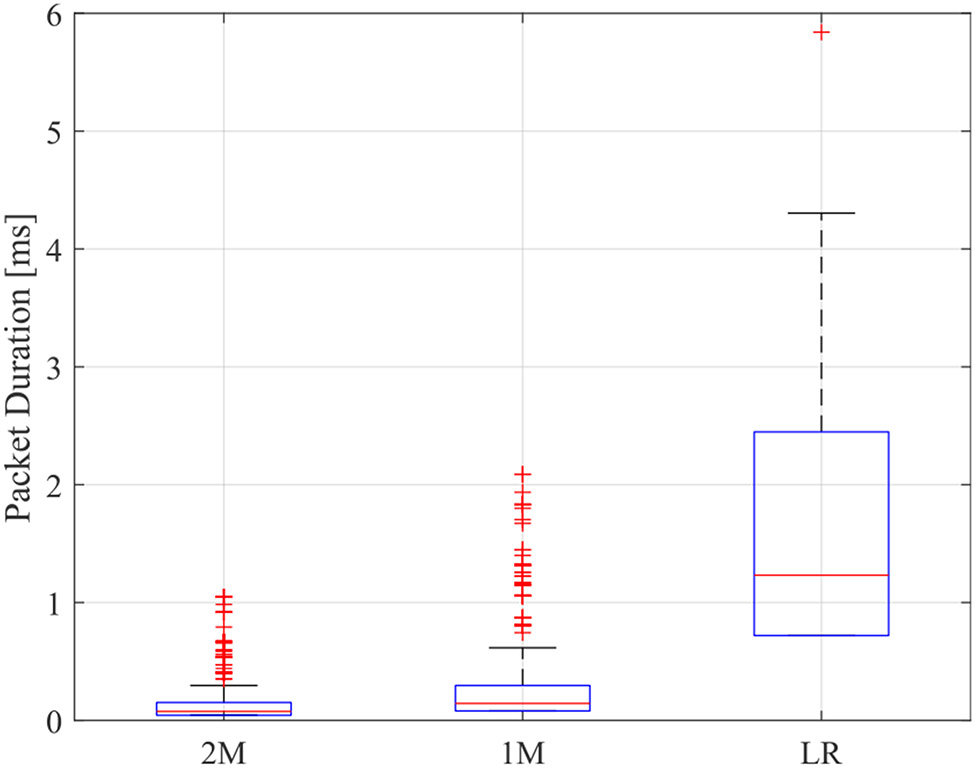
Box plot of packet durations for BLE physical layers from tier 1 scenario.

Performance of the LBT-based network was characterized in terms of the normalized achieved throughput of the PDSCH traffic, which passed CRC check during wireless coexistence tests. Fig. 10 depicts the mean normalized throughput of channels with center frequencies 2412 MHz, 2437 MHz, and 2462 MHz in the three evaluation tiers as function of BLE PHY for each priority class. Measurements are normalized with respect to 43, 55, 55, and 33 Mbps, corresponding to LBT classes 4, 3, 2, and 1, respectively. In general, results reveal that the performance of LBT is hindered in the presence of BLE LR. In contrast, 2M and 1M PHY scenarios demonstrated better outcomes. Furthermore, the figure also denotes that under the same BLE PHY, performance of LBT classes decreased as the access priorities decreased—except for class 1 under LR PHY in tiers 1 and 2 scenarios. Attained normalized throughput appears to surpass that of class 2. As indicated in the previous section, the lower the data rate of the physical layer in BLE, the longer it takes to transmit the same amount of data. Hence, 2M packets occupy the least airtime, followed by 1M and LR PHYs, respectively. Fig. 11 corroborates this observation for the three physical layers of BLE with median durations of 76 *μ*s, 144 *μ*s, and 1.232 ms for 2M, 1M, and LR, respectively. The remarks on the behavior of BLE PHYs as observed on LBT performance are ascribed to the corresponding packet airtime durations. We also note the effect of contention window size and COT pertaining to different LBT access priorities. Although low priority frames occupied the channel for longer times, they exhibited a wider contention window than higher priority classes. These two parameters, along with the duration of BLE packets being sent, create the dynamics behind the impact on the LBT network. Classes 1 and 2 have the same COT, per Table 1, although class 1 bears a maximum contention window size of 1024, compared to 64 for class 2. In a congested radio environment, as is the case in tier 1, class 1 avoids colliding with the coexisting RAT due to longer back-off periods, thus, improving achieved throughput.

**FIGURE 12.**
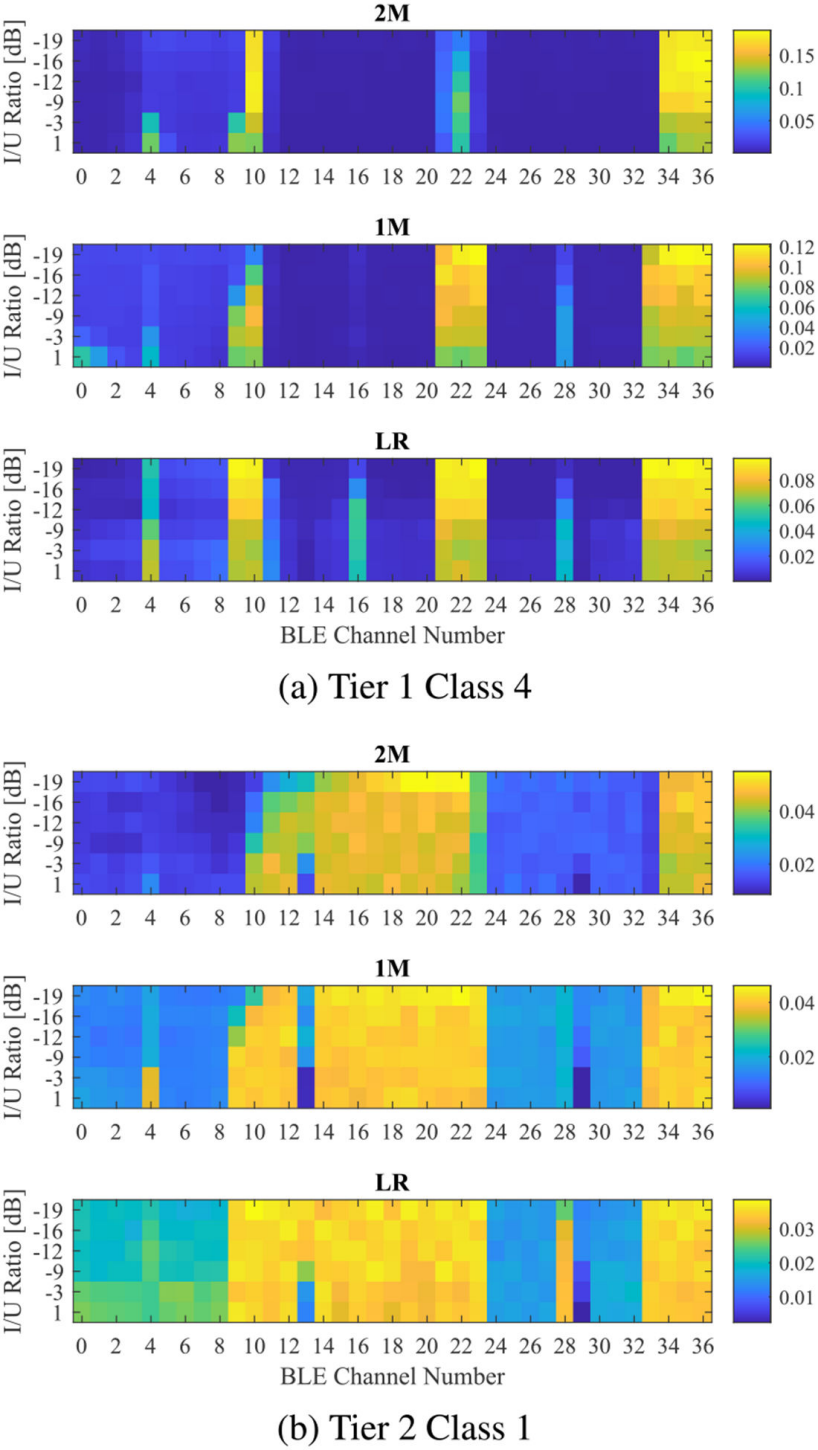
BLE channel histogram as a function of I/U ratio.

Further analysis of BLE data revealed insights on its channel activity. Fig. 12a and 12b illustrate the BLE channel histograms as a function of I/U ratio for the three physical layers under tier 1 class 4 and tier 2 class 1 interferers, respectively. The figures indicate that BLE utilized channels within LBT’s 20-MHz bandwidth (i.e., channels 1, 6, and 11) when temporal activity is low. This is demonstrated by the light blue squares in Fig. 12b corresponding to class 1, compared to darker squares in Fig. 12a corresponding to class 4. However, histogram data highlights that BLE used channels 4, 16, and 28 frequently within LBT transmissions, especially with physical layers 1M and 2M, and high TX powers. Those channels coincide with the DC null subcarrier of OFDM waveform corresponding to LBT center frequencies 2412 MHz, 2437 MHz, and 2462 MHz. This is related to the channel selection algorithm of BLE which determined to use those channels since no transmission occurred at that subcarrier in the center of the band. Nevertheless, this phenomenon caused interference with LBT, which translated into reduced throughput performance as corrupted packets did not pass the CRC check at the receiver side.

## VI. DISCUSSION

Wireless coexistence evaluation is important in several applications including medical devices. Because the healthcare industry is increasingly incorporating wireless connectivity in the end-user equipment, a number of use case scenarios are emerging in this domain like remote pervasive monitoring, healthcare for rural areas, and mobile health using wearables [45]. Such scenarios can employ different wireless technologies within proximity where coexistence issues might arise. Given the risk to patients associated with the delay or disruption of a wireless communication link, evaluating the device for wireless coexistence was recommended in the U.S. Food and Drug Administration (FDA) guidance document on radio frequency wireless technology in medical devices [15]. The ANSI C63.27 standard [16] was developed to address this evaluation. It provides manufacturers with detailed procedures to evaluate the coexistence of a given functional wireless performance (FWP) against recommended test interferers. The tier of evaluation is determined based on the risk category associated with the FWP per AAMI TIR 69 [39]. TIR 69 specifies four risk categories for the wireless function of medical devices; these are listed in Table 4. Annex A of ANSI C63.27 details normative guidelines for some of the most common RATs and frequency bands (e.g., a Bluetooth DUT operating in the 2.4 GHz ISM band is recommended to be tested with IEEE 802.11n signals as an in-band interferer, as well as LTE signals on the lower and upper adjacent bands). A revised draft of C63.27 has been developed and is going through the balloting process of ANSI Accredited Standards Committee C63. The revision included addressing the coexistence of LTE-LAA and Wi-Fi systems in the 5 GHz band [46]. Since LBT-based 5G NR operation in the 2.4 GHz unlicensed spectrum (i.e., commonly known as 5G NR-U) has been recently identified in technical report TR 38.889 [10], it is reasonable to consider the coexistence characteristics of such systems and how to include them in the C63.27 test protocol. Accordingly, the presented experiment in this article, along with the results discussed in Section V, can be used to devise comparable test plans for a BLE 5 DUT and LBT-based interferer in the 2.4 GHz ISM band (e.g., the choice of LBT class and BLE PHY).

Equally important, findings of coexistence testing could inform the design, development, and deployment of LBT-based and/or BLE 5-based applications within the same vicinity. Depending on the DUT’s FWP, pass/fail criteria can be defined and tested under a given operational condition (e.g., a throughput threshold, delay tolerance, etc.). For example, if an application requires an achieved throughput no higher than 100 kbps, BLE PHYs 2M and 1M may be used if the intended environment is less likely to exhibit a busy spectrum band (i.e., similar to tier 1 scenarios where three interferers occupy the 2.4 GHz band). On the other hand, applications where long-distance links and resilience against interference are desired, LR PHY accommodates such needs by virtue of its error correction method at the expense of lower nominal throughput and higher interframe delays compared to the other two BLE physical layers. It is worth mentioning that in more relaxed conditions, where LBT might be serving low traffic applications, the time-domain channel utilization of the unintended signal (i.e., LBT in this case) is lower than the case of the assumed full buffer mode in this work. Hence, the mutual impact on both coexisting networks is alleviated since that would increase the chance for BLE to access the shared medium without colliding with LBT’s traffic. Similarly, LBT-based DUTs might employ results discussed herein to draw on the impact of BLE interference on system performance under different channel access priorities. Such assessments could be coupled with analytical techniques similar to the one reported in [30] to take into consideration the effect of same-technology devices with different combinations of priority classes.

## VII. CONCLUSION

The 3GPP is developing the fifth generation of wireless broadband technology while identifying the unlicensed spectrum as a principal item on the plan of action. Listen-Before-Talk (LBT) has been recognized as the starting development point for the channel access scheme of future 5G NR-U networks. Recent technical reports suggest that all sub-7 GHz unlicensed spectrum are targeted for 5G NR-U, including the 2.4 GHz ISM band. The operation of LBT in the 2.4 GHz raises new wireless coexistence concerns with incumbent RATs that have not been addressed yet. The LBT-based LTE-LAA and Wi-Fi wireless coexistence in the 5 GHz band has been studied extensively in literature, and observations made therein could be extended to the 2.4 GHz spectrum. Notably, BLE is another prominent wireless standard that faces coexistence issues with LBT systems. This article reports the mutual impact of BLE 5 and cellular LBT coexisting systems by means of empirical evaluation. Effects of various parameters of both RATs (e.g., LBT’s channel access priorities and BLE’s physical layers) were investigated. Results were presented in terms of achieved throughput and IFS delay under different parameter combinations and ANSI C63.27 evaluation tiers. It was found that normalized BLE throughput drops as the intended-to-unintended signal ratio decreases and LBT classes exhibit a diminishing effect as the class priority descends. Furthermore, coded BLE PHY (LR) demonstrated less susceptibility to interference in relaxed conditions (e.g., single-channel interferer) compared to 2M and 1M BLE PHYs. Delay analysis indicated that LR sustains longer average gap times than the other two physical layers even though it showed less sensitivity to interference in that regard. On the other hand, results demonstrated that low data rate PHYs hinder the LBT performance as they correspond to longer airtime durations. Outcomes of coexistence testing could help in characterizing and enhancing the operation of a BLE 5 device when sharing channel resources with a future LBT-based system in the 2.4 GHz ISM band.

We believe this work opens the door for further studies to address the concern of coexistence of cellular LBT systems with BLE 5 and other 2.4 GHz ISM band RATs, such as ZigBee. Measurements and findings reported herein could be expanded in the future by investigating more realistic settings (e.g., the effect of multipath). Influence of other connection parameters (e.g., BLE connection interval and packet size) are also important for a complete understanding of the performance and their impact on the coexisting RAT. The effect of heterogeneous LBT channel access priorities in the same channel and across different channels on the neighboring BLE network is an interesting direction of research that is left for future work.

## Figures and Tables

**TABLE 1.** Channel access priorities according to ETSI LBT.

Class	P0	CW_min_	CW_max_	COT [ms]
4	1	4	8	2
3	1	8	16	4
2	3	16	64	6*
1	7	16	1024	6*

* can extend to 8 ms if transmission includes 100 *μ*s pauses

**TABLE 2.** Summary of BLE 5 physical layers.

PHY	1M	Coded		2M
		S2	S8	
Symbol Rate	1 Msym/s	1 Msym/s	1 Msym/s	2 Msym/s
Data Rate	1 Mbps	500 kpbs	125 kbps	2 Mbps
Error Detection	CRC	CRC	CRC	CRC
Error Correction	No	Yes	Yes	No
Requirement	Mandatory	Optional	Optional	Optional

**TABLE 3.** BLE 5 configuration parameters.

PHY	2M, 1M, LR (Coded)
Tx Power	−12, −8, −4, 0, 4, 8 dBm
ATT MTU Size	247 Bytes
Transfer Size	1024 KB
Connection Interval	7.5 ms

**TABLE 4.** AAMI TIR69 risk categories.

Category	Risk and result of failure, disruption, or delay of wireless communication
Category A	High Risk Level: could result in death or serious injury
Category B	Medium Risk Level: could result in injury or impairment requiring professional medical intervention
Category C	Low Risk Level: could result in temporary injury or impairment not requiring professional medical intervention
Category D	No Significant Risk Level: could result, maximally, in inconvenience or temporary discomfort
